# Protein aggregation drives cell aging in a size-specific manner in *Escherichia coli*

**DOI:** 10.1128/mbio.02050-25

**Published:** 2025-12-16

**Authors:** Linda Wagener, Arpita Nath, Murat Tuğrul, Abram Aertsen, Ulrich K. Steiner, Audrey M. Proenca

**Affiliations:** 1 Freie Universität Berlin, Institute of Biology 98893, Berlin, Germany; 2 Department of Microbial and Molecular Systems, KU Leuven 366791 , Leuven, Belgium; Michigan State University, East Lansing, Michigan, USA

**Keywords:** aging, protein aggregation, cell asymmetry, microfluidics, single-cell microscopy

## Abstract

Aging, the decline in physiological function over time, is marked by the intracellular accumulation of damaged components. It can be attributed to trade-offs between organismal maintenance and the generation of high-quality offspring, where the parent retains damage upon reproduction and produces rejuvenated descendants. This occurs even in bacteria, such as *Escherichia coli*, which asymmetrically partition aggregates of misfolded proteins upon division. However, there is conflicting evidence on the fitness impact of protein aggregates, ranging from detrimental effects to enhanced stress survival. Here, we show that the decisive factor driving growth decline in *E. coli* is not the presence of an aggregate, but the fraction of the intracellular space it occupies. By following single-cell *E. coli* lineages expressing fluorescently labeled DnaK chaperones, we quantified damage accumulation and partitioning across generations in microfluidic devices. We found that the diameter of aggregates increases at linear rates, and cell growth declines as a function of the intracellular space lost to damage. At the same time, however, the mother cell undergoes a progressive enlargement that accommodates the growing aggregate. This could be regarded as a compensatory mechanism, allowing the mother to sustain stable growth despite the continuous accumulation of damage and resulting in the emergence of a morphological asymmetry between mother and daughter cells, challenging the long-standing assumption that *E. coli* divides symmetrically. Our findings point to a more complex role of protein aggregation, with implications for our understanding of the cellular mechanisms underlying aging, as well as its evolutionary origins.

**IMPORTANCE:**

Among the simplest organisms known to age, *Escherichia coli* bacteria suffer a functional decline as misfolded proteins accumulate into aggregates retained by the mother cell upon division. This mechanism places the loss of proteostasis as a conserved hallmark of aging. However, subsequent studies found no deleterious effects of harboring aggregates. By quantifying single-cell fitness and damage dynamics, we found that it is not the mere presence of an aggregate that drives a fitness decline, but the intracellular space it occupies. Yet, aging cells undergo a gradual enlargement that could allow them to sustain stable growth despite harboring intracellular damage. Cell enlargement thus emerges as another cross-domain aging phenotype, but with curiously opposite effects: protective in bacteria, whereas generally deleterious in eukaryotes. Our findings, therefore, offer a connection between damage dynamics, aging, and cell size regulation at the single-cell level, while tracing new parallels between bacterial and eukaryotic aging.

## INTRODUCTION

Organisms age due to a decline in fitness over time, losing physiological integrity and becoming increasingly more vulnerable to death (1, 2). Aging can be attributed to a trade-off between reproduction and somatic repair, in which the maintenance of the germline comes at the cost of an increased damage accumulation by a “disposable” soma (3). Although unicellular organisms lack a differentiation between soma and germline, similar processes of asymmetric damage partitioning have led to the emergence of aging in yeast (4), algae (5), and bacteria (6, 7). These single-cell organisms can provide a system where the progression of aging hallmarks can be observed and quantified *in vivo* (1), thus offering a means to elucidate the origins of organismal aging at the cellular level.

**Fig 1:**
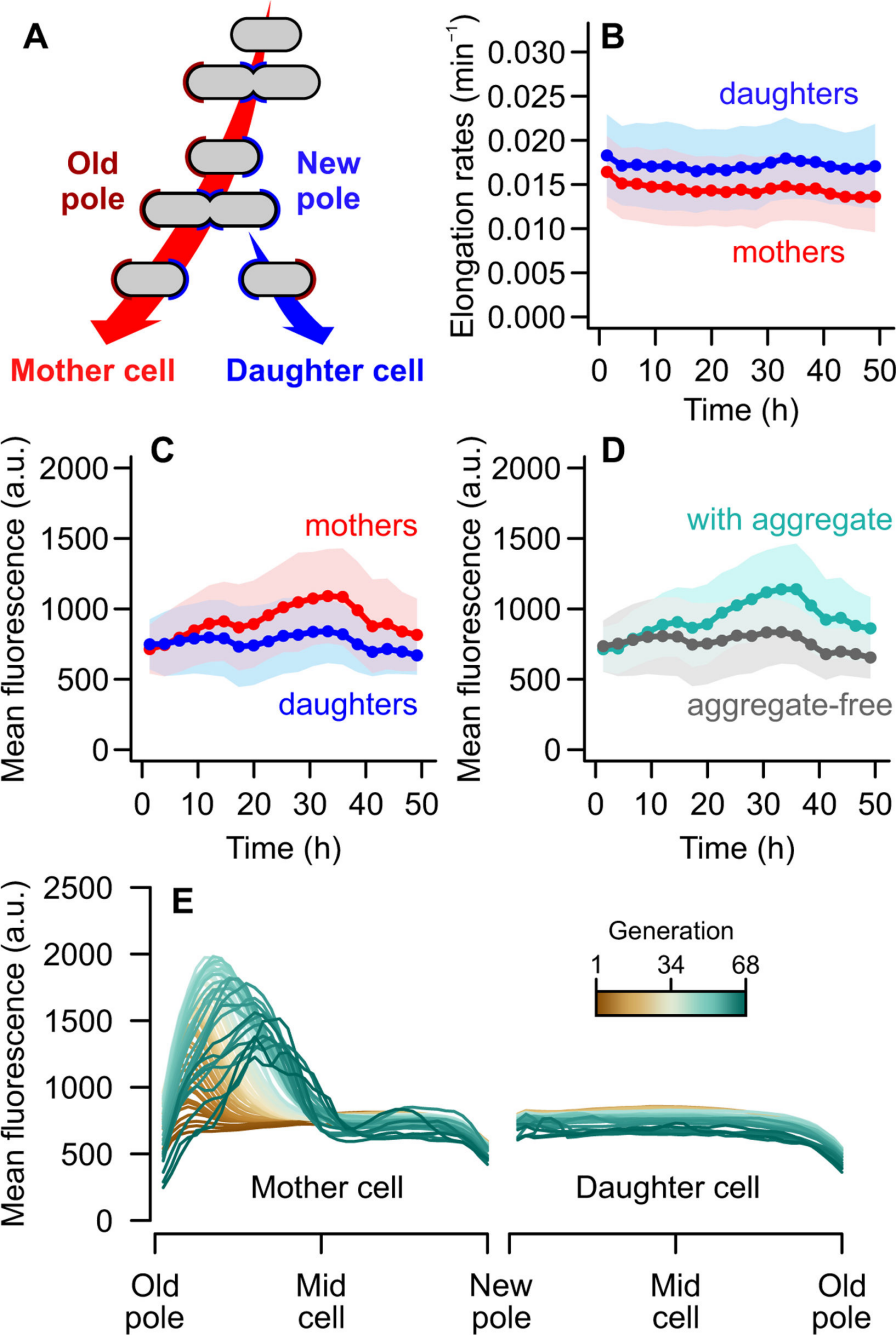
Fluorescence increases over time in mother cells due to aggregates. (**A**) Schematic representation of bacterial lineages according to asymmetric pole inheritance, where mother cells retain an old pole, and daughters inherit a new pole formed at the previous fission site. (**B**) When grown in the constant conditions of a microfluidic device, mothers and daughters showed stable yet asymmetric growth over time (see also Table S1). (**C**) Mean fluorescence, on the other hand, increased over time in mother cells but not in daughters. (**D**) When the population was divided according to the presence/absence of aggregates, they showed a similar increase in mean fluorescence over time as (**C**), suggesting strong overlap of mother-cell and aggregate-bearing subgroups. (**B–D**) Bins = mean ± SD. (**E**) Mean DnaK-msfGFP fluorescence profiles of mother (left) and daughter cells (right) from old to new pole. Each line represents the average transect for a given generation over a normalized cell length. The mother shows increased GFP levels, indicating protein aggregation near the old pole.

Symmetrically dividing prokaryotes, such as the rod-shaped model bacterium *Escherichia coli*, were once thought not to exhibit the aging phenotype. However, despite splitting into two cells that are considered morphologically and genetically identical, *E. coli* displays a deterministic physiological asymmetry (7–9). Upon each division, the two resulting cells inherit either an old pole, conserved across generations, or a new pole, synthesized during the previous fission event (Fig. 1A). Compared to new-pole cells, those inheriting an old pole for multiple generations exhibit slower growth rates, decreased offspring production, and increased mortality rates (7, 10). The continuous old pole lineage can thus be regarded as a mother cell that ages across generations and that produces rejuvenated daughters with each division. Because this asymmetry presumably arises through the uneven partitioning of intracellular damage (8, 11, 12), it can be regarded as akin to a soma-germline delineation (13). Models on asymmetry in single-cell organisms underpin these findings by showing that asymmetrical segregation of damage is likely to evolve as a strategy to increase fitness in the face of high levels of cellular damage (14–16). While these models provide a mechanistic understanding of cellular aging, they rely on a few empirical accounts on the nature of this damage, its partitioning dynamics, and physiological impacts.

The accumulation of misfolded proteins is widely regarded as a source of age-associated intracellular damage. Sources of proteotoxic stress can cause proteins to change from their native conformation to a misfolded one, rendering them inactive or even toxic to the cell. The misfolding of a protein can expose its hydrophobic portions, leading it to bind to other proteins and thereby interfere with their functioning (17). This disruption of proteostasis can lead to the formation of insoluble protein aggregates within a cell. In mammals, the increased accumulation of misfolded proteins and decreased repair capacity is considered an aging hallmark (1), often leading to the development of aging-related diseases, such as Parkinson’s and Alzheimer’s (18). In a striking parallel with eukaryotic processes, *E. coli* aging has been attributed to protein aggregation. Misfolded proteins, which can appear anywhere within the cell, tend to cluster together. If such aggregates become too large to diffuse through the nucleoid (12, 19, 20), they can become stuck at the cell pole as it is successively inherited across generations, thus being preferentially retained by the mother cell (8, 11). Since the presence of aggregates hinders diffusion of other intracellular components (19), it might also cause the mother cell to inherit fewer ribosomes (21) and newly synthesized proteins (22). Daughter cells, in contrast, inherit a larger share of “good” components along with smaller damage loads, leading to improved rates of growth and gene expression (23).

Proteostasis is essential for cellular functioning, and protein repair is key for healthy cell growth. The protein repair machinery of bacteria acts to degrade or refold misfolded proteins, thereby preventing aggregation or disaggregating already formed aggregates (24). As such, protein aggregation mainly occurs when a cell accumulates misfolded proteins at a faster rate than it can repair (11), at which point *E. coli* cells exhibit increasing amounts of polar aggregates. Computational analyses suggest that aggregation can be advantageous in the face of stress, as it allows for the asymmetric partitioning of intracellular damage between aging mothers and rejuvenating daughters (25). If the same damage loads were to be symmetrically partitioned, the preservation of cellular functions would likely require a larger investment into repair activities.

Nonetheless, while it is known that protein aggregates form in response to stress, their correlation with aging phenotypes has been questioned by recent studies. The observation of large aggregates in maternal old poles has been attributed to the choice of fluorescent marker in earlier research, which used the small chaperone Inclusion Body Protein A (IbpA) labeled with yellow fluorescent protein for damage quantification (8, 26). When the reporter was exchanged for a monomeric superfolder green fluorescent protein (msfGFP), heat shock-induced aggregates had no negative impact on fitness (27). Similarly, *E. coli* lineages grown in unstressed conditions did not accumulate polar aggregates, despite consistently exhibiting a physiological asymmetry between mothers and daughters (28). The presence of an aggregate, however, does not always imply a fitness decline; once their formation is induced (e.g., through exposure to non-lethal stress), they can have a protective effect against further heat shock (27) and promote antibiotic persistence (29).

The observation of an age-related loss of proteostasis in prokaryotes has framed this process as a conserved aging hallmark among cellular organisms (30). It is therefore imperative to elucidate the recent conflicting evidence on the correlation between protein aggregation and bacterial aging. To address this, we quantified the accumulation of misfolded proteins using DnaK, a heat-shock protein that plays a central role in protein quality control, acting both to refold soluble misfolded proteins (31) and to disaggregate protein aggregates (11, 17). As a damage marker, most previous studies have focused on IbpA (8, 12, 27), which co-localizes with misfolded proteins. DnaK, in contrast, reports not only the localization of damage but also indicates that it is being actively repaired by the cell in an energy-consuming process (11, 32). Quantifying the accumulation of fluorescently labeled DnaK, we found that protein aggregates accumulate in maternal old poles over generations, even as mother cells reach the state of long-term growth stability that is commonly observed within microfluidic devices (9, 28, 33). By itself, the presence of a polar aggregate did not lead to lower elongation rates. Instead, we found that the main determinant of aging is the relative size of the protein aggregate, compared to the total length of a cell. We found that aging also correlates with an increase in maternal cell size, which seems to buffer the effect of damage accumulation and allows the cell to maintain stable elongation rates despite harboring large clusters of misfolded proteins. We thus propose that protein aggregation is a hallmark of bacterial aging, driving a progressive functional decline in a size-dependent manner.

## RESULTS

### Mean concentration of misfolded proteins increases with age

To evaluate whether protein misfolding and aggregation act as a driver of bacterial aging, we quantified their accumulation at the single-cell level. For this, we used *E. coli* cells expressing DnaK-msfGFP as a reporter for both the subcellular localization of misfolded proteins and the ongoing repair activity that is expected to be energy-costly (11). We ensured stable conditions by culturing cells in the mother machine microfluidic device (33), containing thousands of growth wells that receive a continuous supply of nutrients. The closed end of each well traps the cell inheriting an old pole with each division, which can be regarded as the mother cell. The opposite pole, synthesized during the previous fission, is considered a new pole that is inherited by the daughter cell. While the device traps mother cells for hundreds of generations, daughters are lost into the flow channel after a few divisions. Here, we followed populations over 48 h, measuring growth physiology (cell length, elongation rates, division intervals) and misfolded protein accumulation (fluorescence distribution) in mother cells over time, and in daughter cells over their first cell cycle. Overall, our analysis encompasses 487 cell lineages expressing DnaK-msfGFP across a total of 18,756 division events.

We first evaluated how elongation rate is shaped by time, divisional asymmetry, and expression of the fluorescent fusion (Fig. 1B). For this, we considered the reporter strain either individually or in comparison with a control wild-type strain (Fig. S1), evaluating them through generalized additive models (GAMs; statistical models provided in the Supplementary Text). In both cases, daughter cells grew faster than their mothers, displaying a higher intercept when elongation rates were considered across time (Fig. 1B). This asymmetry accounted for ~7.7% of the deviance in the data (Table S1 to S3). While accounting for changes in asymmetry over time improved the model fit, this interaction accounted for <0.2% of the deviance in elongation rates. The reporter strain, therefore, exhibited the expected properties of *E. coli* cells growing in a mother machine device (9, 28, 33), showing stable growth with deterministic differences between mothers and daughters.

To determine whether aging correlates with the accumulation of misfolded proteins, whether soluble or aggregated, we measured the mean DnaK-msfGFP fluorescence after each division. We found that the concentration of misfolded proteins in mother cells increased over time, which was contrasted by no relevant increase in daughter cells (Fig. 1C). We verified this statistically through the comparison of multiple GAMs (Table S4). This analysis showed that asymmetry leads to distinct mean fluorescence levels in mothers and daughters, which varied independently across time. Considering both this mean effect and the interaction with time, asymmetry accounted for 7.3% of the deviance in mean fluorescence. As an individual effect, mean changes across time explained another 7.1% of the deviance in the data. The variance among maternal lineages was largely meaningful (deviance explained = 42.8%) for DnaK-msfGFP levels, suggesting that once a lineage reaches a certain concentration of misfolded proteins, the mother cell is likely to retain them over successive divisions. Later fluctuations in mean fluorescence, observed in both mothers and daughters after 36 h, are likely due to the loss of tracked cells over time.

The asymmetric retention of damage depends on both the formation of protein aggregates and their retention in maternal old poles. To investigate the relationship between asymmetry and protein aggregation, we repeated our analysis while splitting the population according to the presence of fluorescence clusters (Fig. 1D), independently of polar age. The resulting difference between aggregate-bearing and aggregate-free cells largely reflected that of mother-daughter asymmetry (Table S5), explaining a total of 8.15% of the deviance in mean fluorescence. This similarity suggests that the maternal increase in DnaK fluorescence was due to the accumulation of protein aggregates, which are mostly absent among daughter cells.

Next, to determine whether these aggregates were associated with maternal old poles, we quantified the intracellular distribution of DnaK-msfGFP (Fig. 1E). We traced the mean fluorescence profile along the length of each cell immediately after division and estimated the effect of time and the distance from the old pole on fluorescence levels (Table S6). While the profiles in Fig. 1E depict the average measurements for all cells at a given generation (where *n* = 487 profiles at generation 1), statistical analyses were performed on individual transects. For mother cells, we found that fluorescence levels changed across the cell axis, with the shape of this profile changing over time (Table S6). Once we accounted for this interaction between the subcellular localization and time, including individual effects of time on mean fluorescence, resulted in little improvement in the deviance explained by the models. This indicates that mother cells undergo a localized accumulation of misfolded proteins over generations, while showing small changes to the levels of dispersed fluorescence. Daughter cells, on the other hand, had slight changes in mean fluorescence levels over time and across the cell length, but no interaction between these factors. Daughters may contain occasional aggregates with no particular subcellular localization bias, and which are not visible in average profiles.

Taken together, these data indicate that mother cells retain large clusters of damaged proteins over generations, whereas daughters are usually born free of protein aggregates. Beyond a passive accumulation of damage in maternal old poles, this asymmetric inheritance implies an active process: since DnaK acts to refold and disaggregate damaged proteins, consuming ATP in the process, the data in Fig. 1 also raises the possibility of an asymmetric allocation of resources where mother cells might show a greater investment into repair activities.

### Size of maternal aggregates increases over time

**Fig 2:**
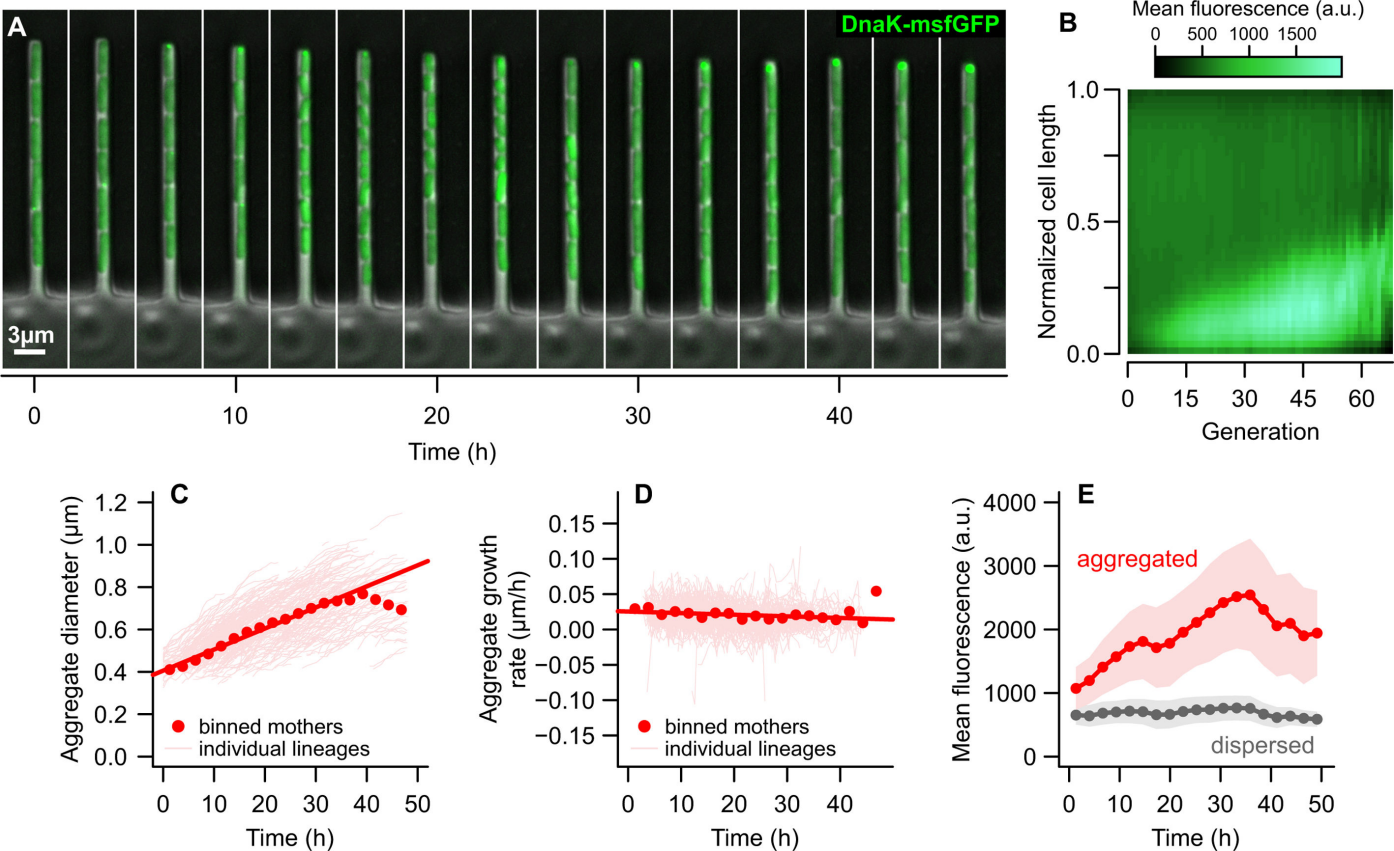
Dynamics of damage accumulation within mother cells. (**A**) Time-lapse images of a cell lineage growing within the mother machine device. The mother cell, at the closed end (top), accumulates DnaK-msfGFP into fluorescent foci, while its daughters (toward the open end of the growth well) exhibit dispersed fluorescence. (**B**) Heatmap of the mean fluorescence of mother cells across generations, showing that the concentration of DnaK-msfGFP increases in the old poles due to the formation of aggregates. (**C**) Pooling all aggregate-bearing mothers, we observed that the diameter of protein aggregates increases at a linear rate. over time (solid red line, R^2^ = 0.384, *P* < 0.001). (**D**) Aggregate diameters changed at a constant rate from one generation to the next, for rates individually estimated for each mother lineage. (**E**) DnaK-msfGFP concentration in the form of aggregates or dispersed fluorescence in aggregate-bearing mothers. While aggregates increased in size and fluorescence intensity over time, there was little variation in the concentration of misfolded proteins in the rest of the cell. Thus, mother cells accumulate damage at a constant rate as they age, corresponding to the clustering of misfolded proteins into polar aggregates. (**C–E**) Bins = mean ± SD.

To further investigate the dynamics of protein misfolding and aggregation over generations, we quantified the growth of protein aggregates in maternal lineages. For this, we evaluated fluorescence transects showing the distribution of DnaK-msfGFP throughout the cell’s longitudinal axis after each division. We detected aggregates as fluorescence peaks on each profile (see Materials and Methods for details), quantifying their diameter, intracellular position, and mean intensity. As suggested by the average transects in Fig. 1E, we observed increasingly large peaks associated with maternal old poles (Fig. 2A). The aggregates showed an average distance of 0.22 µm from the old pole (Fig. 2B), which represents 7.9% of the mean length of a mother cell at the beginning of a cell cycle (2.82 µm). In total, mother cells harbored aggregates on 66.9% of the observed division events. Daughter cells, on the other hand, showed homogeneous levels of intracellular fluorescence (Fig. 1E, right panel), with aggregates being detected only on 3.6% of the quantified transects.

As suggested by the fluorescence profiles in Fig. 1E, maternal aggregates grew larger over time (Fig. 2C; Table S7). Evaluating the growth rate of aggregates across all mother cells, we observed a linear increase in diameters prior to 40 h of exponential growth at a rate of 0.01 µm/h (Size = 0.01 × Time + 0.4, *P* < 0.001). Aggregate growth seems to become non-linear after 40 h, at which point their diameters are expected to reach 0.8 µm—the average width of a cell in our experimental conditions (Fig. S2). To determine whether these growth rates were stable, we estimated the changes in aggregate diameters between consecutive generations for each maternal lineage (Fig. 2D). Although aggregate growth rates fluctuated slightly, any changes over time were negligible (Table S7). By fitting a GAM that evaluated the effect of time and variation among mother cells on aggregate growth, we found that these variables explained 0.09% of the deviance in the data, indicating that aggregates grow in diameter at a stable rate that is subjected to stochastic fluctuations. Interestingly, the linear increase of aggregate diameters with age suggests a buffering effect of aggregation: if we assume that aggregates are spherical, a linear growth in diameter indicates a quadratic change in volume (see Supplementary Text). The quantity of misfolded proteins added to the aggregate would also need to increase quadratically over time, thus implying that a quadratic accumulation of damage only has a linear effect on aggregate diameter growth.

Next, to determine whether mother cells also accumulate non-aggregated misfolded proteins, we quantified the concentration of DnaK in aggregates versus the rest of the cell (Fig. 2E). For this, we determined the intracellular area occupied by fluorescence peaks and the area free of aggregates, classifying the mean fluorescence of the former as “aggregated” and the latter as “dispersed.” We found that, while mother cells accumulated misfolded proteins in the form of aggregates (F = 1,310.87, *P* < 0.001), their levels of dispersed fluorescence showed little change over time (F = 63.72, *P* < 0.001; Table S8). Together, these results indicate that the accumulation of damaged proteins as mother cells age is strongly associated with their deposition into aggregates, whereas the damage dispersed in the remaining intracellular space stays at baseline levels. We corroborate that the formation of polar aggregates across generations allows for the retention of damage by the mother cell, leading to the asymmetric partitioning of misfolded components upon division.

### Dispersed misfolded proteins are more harmful than aggregates

The increase in aggregate size over time contrasts with the stability of bacterial elongation rates across generations, found in our results (Fig. 1B), as well as in many previous studies (9, 28, 33). Whether through a toxic effect of harboring misfolded proteins or through the increased investment of resources into repair, it has been widely assumed that protein aggregation should correlate with slower growth (8, 11). Past models have proposed that mother cells reach a state of growth stability once the intracellular damage load retained after each division becomes constant (15, 26). However, our data suggests that damage levels continue to climb long after mother cells have reached this state of equilibrium (Fig. 1B and 2C). We must therefore investigate whether the accumulation of misfolded proteins correlates with a physiological decline (8) or has no effect on growth (27).

**Fig 3:**
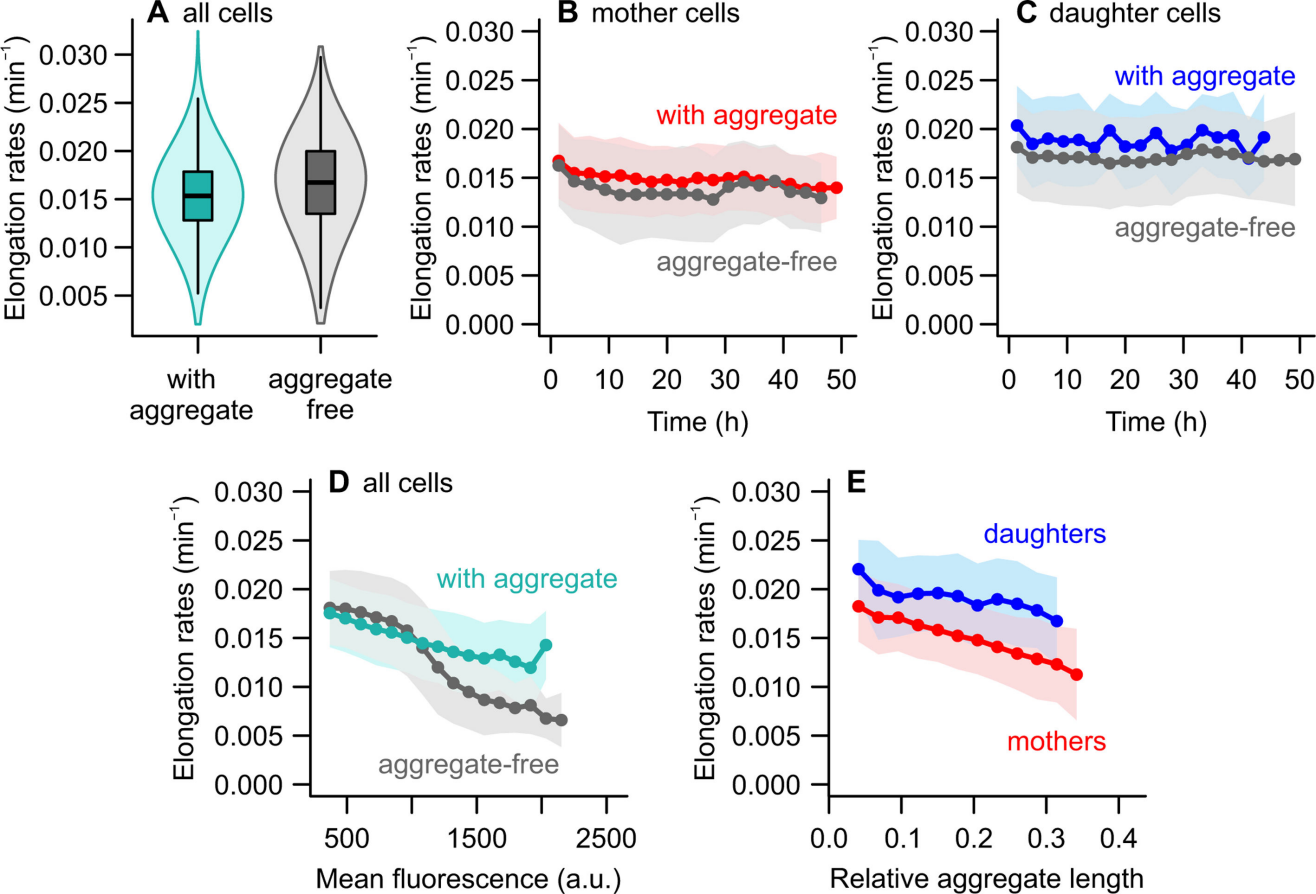
Effect of protein aggregates on cell growth. (**A**) When considering the population as a whole, elongation rates were negatively impacted by the presence of protein aggregates. (**B and C**) Splitting the sample into subpopulations of mothers and daughters showed the opposite effect. Within subpopulations, aggregate-bearing cells had consistently greater elongation rates than aggregate-free cells. (**D**) Intracellular damage loads, as indicated by mean fluorescence, were negatively correlated with elongation rates. High damage concentrations (above 1,000 a.u.) led to a sharp growth decline in cells without aggregates, indicating that dispersed misfolded proteins might be more harmful to the cell at high damage levels. (**E**) The impact of aggregates on elongation rates depends on the intracellular space occupied by this damage, expressed as the relative size of the aggregate when compared to the length of the cell (n_mothers_ = 12,077 observations and n_daughters_ = 676 observations, where each point corresponds to elongation rates measured from birth to division of an aggregate-bearing cell). Bins = mean ± SD.

Considering the whole population, the presence of a protein aggregate reduced elongation rates, albeit effect sizes were small (Fig. 3A): cells bearing an aggregate grew slightly more slowly (0.0156 ± 0.0039 min^−1^) than aggregate-free cells (0.0162 ± 0.0049 min^−1^; t = −24.45, *P* < 0.001) (see also Table S9). However, this difference did not factor in the inheritance of old or new poles upon division. By repeating this analysis while accounting for the asymmetry between mother and daughter cells, we found that this trend was surprisingly reversed (Fig. 3B and C). Aggregate-free cells exhibited slightly slower elongation rates than aggregate-bearing cells in either subpopulation (Table S10; GAM #6: t = 18.12, *P* < 0.001). Most of the difference in elongation rates could be attributed to mother-daughter asymmetry (t = 50.46, *P* < 0.001), whereas interactions between time and asymmetry or the presence of aggregates contributed marginally to explain the deviance in elongation rates (Table S10). Despite the small difference between aggregate-bearing and aggregate-free cells, this suggests that the simple presence of an aggregate does not imply slower growth. Once we account for the underlying asymmetry between mothers and daughters, harboring an aggregate might even provide a slight growth advantage.

This observation appears to stand in contrast with the progressive accumulation of damage among mother cells (Fig. 2C) and the correlation between the inheritance of old poles and slower elongation rates (Fig. 1B). Could it mean that protein aggregation has a protective effect against damage accumulation? To investigate this, we evaluated the mean fluorescence of all cells in the population. Our time-lapse images suggested that, in some cases, growing cells with no discernible aggregates could nonetheless exhibit a high concentration of DnaK-msfGFP. In both mothers and daughters, a fraction of aggregate-free cells showed a mean fluorescence higher than 1,000 a.u., an intensity threshold associated with the formation of a polar aggregate in most other cases (Fig. 1E): fluorescence foci had an average intensity of 1,799.4 ± 768.3 a.u., with their peaks reaching 2,305.3 a.u., whereas aggregate-free cells showed a mean fluorescence of 776.5 ± 250.5 a.u. By plotting the mean fluorescence of aggregate-free and aggregate-bearing subpopulations against their elongation rates, we observed that a clear distinction emerges for higher DnaK concentrations (Fig. 3D), where cells with high levels of dispersed misfolded proteins had slower growth than those bearing a similar fluorescence level in aggregated form. Although higher fluorescence corresponded to a slower growth in both cases, the effect of intracellular damage on elongation rates was modified by whether the cells had aggregates (Table S11), explaining 17.1% of the deviance in elongation rates.

These findings suggest that an increased damage load in the cell may be less disruptive to cellular growth when it is focused or aggregated, rather than dispersed throughout the entire cell—especially when its concentration reaches higher levels. This raises an interesting perspective on bacterial asymmetry: so far, the asymmetric partitioning of damage has been considered advantageous in the face of high damage accumulation rates, as it might allow for faster population growth than symmetric partitioning (16) and lead to differential stress survival (15, 25). However, these past models considered that a given damage load would have the same effect on cellular growth whether dispersed or aggregated in the pole. If damage aggregation decreases the cost of bearing this intracellular load, the very mechanism of asymmetry might provide a fitness advantage over symmetrically dividing cells, which face the increased cost of dispersed damage.

### Cell aging is determined by the relative size of aggregates

Recent studies have shown that ribosomes (21) and newly synthesized proteins (22, 23) are differentially inherited by the daughter cell, largely due to the exclusion of these components from maternal old poles by protein aggregates (19). Thus, the advantage of aggregate-bearing cells could derive from the confinement of damage and repair functions to a smaller area near the cell pole, leaving more free space for processes necessary for healthy cell functioning and growth. In other words, the defining feature affecting *E. coli* cell growth might be the amount of aggregate-free space in the cell. We tested this hypothesis by determining the fraction of the cell occupied by an aggregate (i.e., relative aggregate length) and determined its effect on elongation rates as a function of cell pole inheritance (Fig. 3E). We found a negative effect of relative aggregate length on the elongation rates of mother and daughter cells (Table S12), which differed slightly in how they varied with increasing aggregate sizes. This suggests that protein aggregates may drive a physiological decline in mother cells through spatial competition with other cellular processes.

### Morphological asymmetry buffers against physiological decline

A puzzling discrepancy still emerges from our results. The diameter of protein aggregates accumulating in maternal old poles increases over time (Fig. 1E), with harmful effects on cellular growth depending on their relative size (Fig. 3E). Nonetheless, elongation rates remain stable across generations. In a bacterium that divides with morphological symmetry, where the size of the mother remains constant, it is not possible to reconcile these observations. If, however, mother cells were to balance out the space that is lost to aggregates by growing longer, this might allow the cell to sustain the normal functioning of growth and protein synthesis.

**Fig 4:**
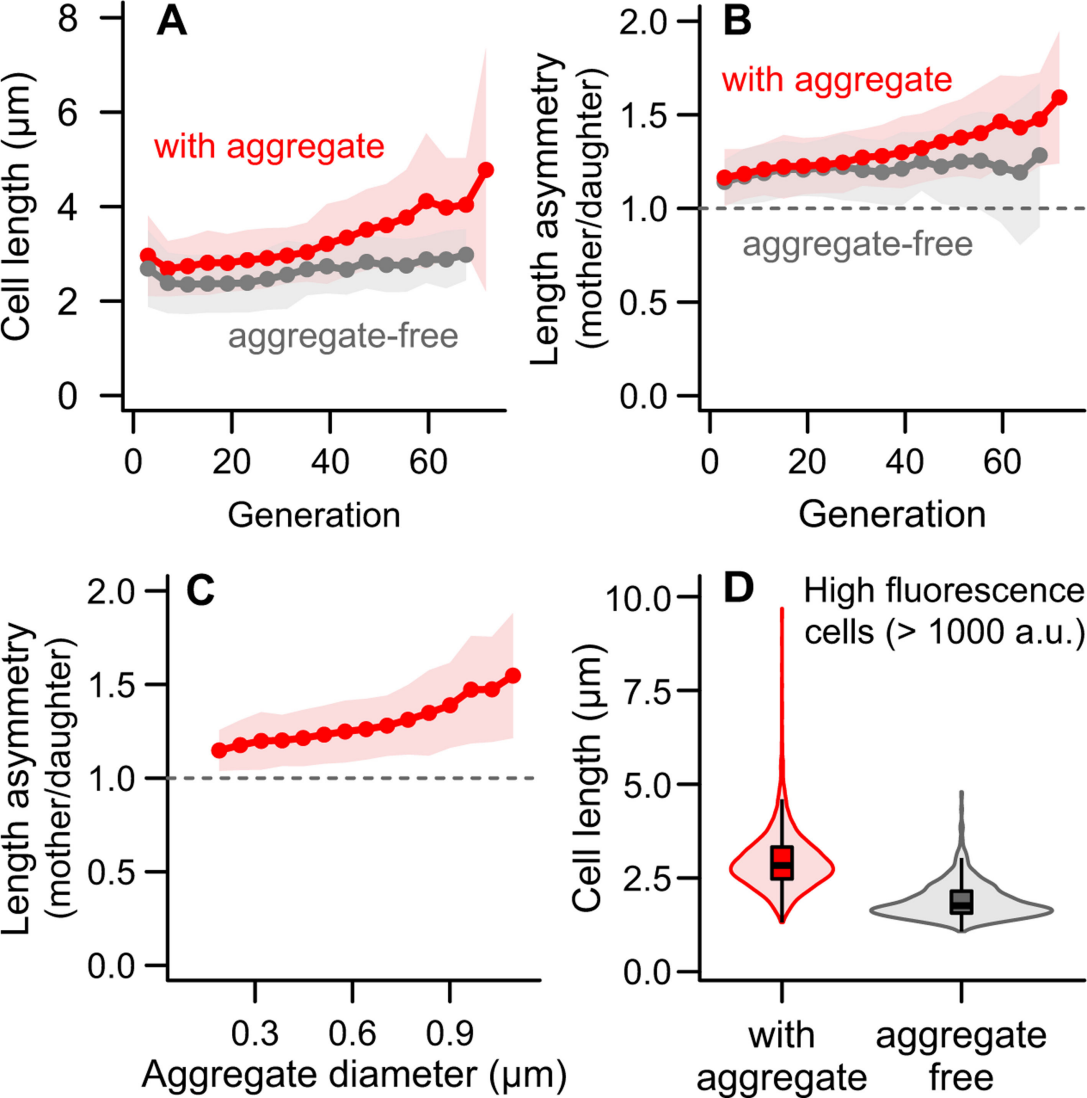
Cell length increases in the presence of protein aggregates, resulting in morphological asymmetry. (**A**) With increasing replicative age, *E. coli* mothers increased in length after division (i.e., start of the cell cycle). The effect of age on length was greater when an aggregate was present. (**B**) Since daughter cells do not undergo the same changes in cell length, a morphological asymmetry emerges as mother cells age. This length asymmetry increased with age in aggregate-bearing cells. (**C**) In divisions where the mother cell carried an aggregate, length asymmetry correlated positively with the size of the aggregate. (**A–C**) Bins = mean ± SD. (**D**) Among mother cells with high DnaK concentrations, those bearing aggregates were longer than those containing only dispersed damage. Without the buffering effect of longer lengths, achieved through morphologically asymmetric divisions, the latter suffer a steeper decline in elongation rates for equivalent concentrations of damage (Fig. 3D).

To test this hypothesis, we evaluated the effects of protein aggregates on the length of mother cells at the start of each cell cycle. We found that cell length increased with replicative age (Fig. 4A), and this effect was modified by the presence of protein aggregates: aggregate-bearing mothers showed a steeper increase in length across generations (F = 426.06, *P* < 0.001) compared to aggregate-free mothers (F = 61.68, *P* < 0.001; GAM #1 in Table S13). These changes in cell length were not accompanied by changes in width, which remained largely stable over time (Fig. S2). Because daughter cells do not undergo such changes in length, a morphological asymmetry between mothers and daughters emerges (Fig. 4B). The length asymmetry becomes more pronounced with each division, a pattern that is shaped by the presence of aggregates in mother cells (F = 334.75, *P* < 0.001; GAM #1 in Table S14). This effect can be attributed to the displacement of the FtsZ fission ring by aggregates (34), leading to off-center divisions. In fact, we found that the larger the diameter of the aggregate, the greater the morphological asymmetry (Fig. 4C; GAM: F = 215.4, *P* < 0.001). We observed the same patterns when considering the effect of aggregates on cell area, since it increases as a function of length (Fig. S3).

These results support the idea that damage competes for intracellular space with components necessary for cell growth and metabolism. Newly synthesized proteins and ribosomes are known to be excluded from areas occupied by aggregates (19) and, consequently, from maternal old poles (21–23). The accumulation of misfolded proteins in the form of aggregates thus hinders cell growth according to the intracellular space occupied by this damage (Fig. 3E). At the same time, however, the formation of aggregates leads to the elongation of the mother cell (Fig. 4A). Because the remainder of the cell has a constant baseline damage level (Fig. 2E), this allows the mother to sustain constant elongation rates (Fig. 1B) even as aggregates increase in size (Fig. 2C). The consequences of lacking this buffering mechanism are best exemplified through a comparison between mother cells harboring high mean fluorescence (Fig. 4D): mothers bearing large concentrations of dispersed damage are significantly smaller than those bearing a similar concentration in the form of aggregates (Table S15), and suffer a stronger fitness decline with the increase in damage loads (Fig. 3D).

Taken together, our results indicate that cell length is affected by the presence and size of protein aggregates accumulated in maternal old poles, resulting in the emergence of morphological asymmetry in aging *E. coli*. Because the physiological impact of damage accumulation depends on the intracellular space limitations, this length increase may therefore buffer mother cells against the harmful effects of protein misfolding, allowing elongation rates to remain constant.

## DISCUSSION

Bacteria age through the asymmetry between mother and daughter cells upon division, mirroring the separation between a soma and germline in multicellular organisms. This asymmetry is usually attributed to the uneven segregation of damaged proteins, but the impact of protein aggregation on the bacterial aging phenotype is clouded by conflicting results (17, 35, 36). The prevalent assumption was that protein aggregates accumulate in maternal old poles over time, leading to a physiological decline (8, 11), but recent findings challenged this perspective by revealing a lack of correlation between aggregates and cell growth (27).

We addressed these contradictory findings, showing that mother cells gather misfolded proteins into polar aggregates that grow progressively larger over generations (Fig. 2). Although mother cells were likely to retain this damage upon division, the sole presence of an aggregate could not explain their lower elongation rates. Instead, we found that the main factor driving growth decline was the proportion of the intracellular space occupied by the aggregate (Fig. 3E). One possible explanation for this is the spatial competition between damage and the protein synthesis machinery. The intracellular organization of these components is influenced by the nucleoid (37), which holds aggregates in the maternal old pole as they become larger (12). Protein aggregates themselves have been shown to hinder the diffusion of other intracellular components (19), displacing ribosomes (21) and newly synthesized proteins (22) toward the new pole. With increasing maternal age, the cell develops an intracellular gradient in gene expression, where the old pole has a lower contribution to the overall metabolism (23). The combination of these recent data with our findings suggests that the relative size of a protein aggregate determines the physiological decline of the mother cell, as it competes with processes that are necessary for cell growth and functioning.

Nonetheless, an unexpected outcome emerged when we considered the underlying age structure of the population. If mothers and daughters were pooled together, cells inheriting aggregates had slower growth—largely because these tend to be mother cells. In contrast, when we considered subpopulations of mother and daughter cells separately, aggregate-bearing cells showed marginally faster elongation rates than aggregate-free cells (Fig. 3). This effect was more pronounced among cells with a high concentration of intracellular damage, suggesting that polar aggregates might be less detrimental to cellular functioning than similar levels of dispersed damage. This implies that protein aggregation can buffer an aging mother cell against a more severe physiological decline with age. One reason for this buffering effect is that a linear growth in aggregate diameters can only be achieved by a quadratic addition of misfolded proteins. Moreover, the formation of polar aggregates has been shown to interfere with the positioning of the FtsZ ring, resulting in off-center divisions (34). This provides a mechanistic explanation for the gradual increase in maternal length as the cell accumulates more damage (Fig. 4), in a process that might mitigate the spatial competition between damaged proteins and other cellular components. The result is the emergence of a morphological asymmetry in *E. coli* division as the mother cell ages, a finding that contrasts with the long-assumed morphological symmetry of these bacteria.

The progressive enlargement of *E. coli* mother cells parallels a characteristic aging phenotype found among eukaryotes. Aging cells experience various forms of intracellular damage that can interfere with the progression of the cell cycle, leading to a halt in replication. Cell growth, however, can continue, producing a change in the DNA:cytoplasm ratio of aging cells (38). Enlarged cells cannot properly scale RNA and protein synthesis functions, and thus suffer a physiological decline. This age-related increase in cell size has been reported across levels of eukaryotic organization, from budding yeast (38) to fibroblasts (39) and stem cells (40), and is even more visible among senescent cells (41). Despite the phenotypic parallel, however, it is important to note that the mechanisms and consequences of this enlargement are widely different from our observations. While in eukaryotes, the size increase is tied to an arrest of cell division, *E. coli* mother cells grow longer through successive off-center division events (34). More importantly, whereas this enlargement is highly detrimental among eukaryotes and reversing it can alleviate aging (40), our results indicate that it can offer a protective mechanism for bacterial cells carrying damaged proteins.

Protein aggregation is a well-observed feature of the aging phenotype, and previous research has shown that it can come with fitness advantages—one being asymmetrical segregation of damage during cell division, ensuring rejuvenated, immortal lineages within the population (14, 26). The separation of damaged components from other cellular processes may not only benefit the offspring but also allow the aging cell to sustain stable growth rates. As mother cells accumulate misfolded proteins in the form of polar-localized aggregates, they compensate for the lost intracellular space by increasing in length. Since this gradual elongation occurs through asymmetric fission, it is justified to assume that these cells continue to divide according to the “adder” model (42), growing by a constant length between divisions. This would allow aging cells to maintain a state of growth stability over generations (9, 28, 33), despite the continuous accumulation of intracellular damage. For *E. coli*, this could mean that aging may not present as a steady growth decline but rather as a prolonged period of stable growth accompanied by focused damage accumulation, which eventually results in rapid decline and death when damage amounts have reached a lethal level. The existence of such mortality thresholds has been suggested by mathematical models (15, 43) and even estimated from experimental doubling times (26), but its direct quantification remains elusive.

Our findings provide a framework through which protein aggregation operates in bacterial lineages. Increasing damage loads accumulate in mother cells, but their detrimental effects can be buffered by a corresponding increase in length that allows for stable growth. These results, however, do not explain all mechanisms of bacterial aging. An aggregate-free mother cell still grows more slowly than its daughter, and an aggregate-bearing mother has lower elongation rates despite being buffered by a length increase. We can hypothesize that mother cells show decreased growth not only due to harboring more damage, which they can compensate through increased length, but also due to the repair investment required to prevent the growth of protein aggregates from outpacing the lengthening of the cell. In addition, further aging mechanisms not linked to the accumulation of misfolded proteins might contribute to maternal aging, which might not be surprising given the known complexity of the aging process even in simple unicellular organisms. Studies performed on *Caulobacter crescentus*, for example, suggest that bacteria might display other forms of aging even when the segregation of protein aggregates is symmetric (44). By demonstrating how growth physiology and protein aggregation dynamics interact along the aging process, we provide insights into the underlying mechanisms that drive senescence and asymmetry at the single-cell level. Besides offering guidance for future studies on the complex and interacting mechanisms of protein misfolding, aggregation, and repair, our findings thus contribute to revealing fundamental mechanisms of aging.

## MATERIALS AND METHODS

### Bacterial strains and growth conditions

*E. coli* strains MG1655 *∆lacY dnaK-msfGFP* and MG1655 *∆lacY* (wild type) were developed at KU Leuven, Department of Microbial and Molecular Systems, for a previous study (27). *E. coli* MG1655 *∆lacY dnaK-msfGFP* expresses a DnaK-msfGFP (monomeric superfolder GFP) fusion protein, which allows for the quantification of aggregate formation. Bacterial stocks were streaked into fresh LB plates, and a single colony was inoculated into 5 mL M9 minimal medium (1× M9 salts, 2 mM MgSO_4_, 0.1 mM CaCl_2_) supplemented with 0.4% glucose and 0.2% casamino acids, where it was grown overnight at 37°C with agitation. Before each experiment, 150 µL of the overnight culture was diluted into 15 mL of fresh medium and grown for 2 h at 37°C. For inoculation into microfluidic devices, the medium was supplemented with 1 µg/mL propidium iodide (PI) and 0.075% Tween 20 (Sigma-Aldrich). PI facilitates the detection of cell lysis by intercalating with nucleic acids, and Tween 20 was used to prevent cell adhesion inside the device. Throughout the microfluidics experiments, temperatures were kept constant at 37°C in a microscopy incubation chamber (PeCon TempController 2000-1). This temperature was chosen to reflect the natural gut environment of *E. coli* bacteria and to avoid the stochastic damage accumulation and increased mortality induced by higher temperatures (10, 26).

### Microfluidic device design and fabrication

The mother machine (33) is a microfluidic design that allows for the capture of individual bacterial cells inside narrow growth wells, while being fed fresh culture medium provided by wide flow channels. This allows for the capture of time-lapse images of individual cells growing in the exponential phase over several generations. Cells at the closed end of the wells are considered “mothers” because they inherit the old pole in every division. Their new pole siblings, which get washed out of the well into the flow channels over time, are referred to as daughters. Our microfluidic design contained four parallel flow channels (1 cm × 80 μm × 10 μm) with inlet openings on one side and outlets on the other. At these inlets and outlets, metal connectors were connected to Tygon tubing (ND 100-80, Saint-Gobain, Darwin Microfluidics) to feed M9 media into the device and remove cells that had been washed out of the wells. Each flow channel contains 1,000 growth wells (25 × 0.8 × 1.2 µm) that are long enough for mothers not to get washed out too easily, while being short and wide enough for sufficient growth medium to reach mother cells, as reported by previous studies (33, 45).

We fabricated chips by pouring Sylgard 184 polydimethylsiloxane (PDMS, Dow Corning, USA) into epoxy molds that had been cast from original laser-etched wafers. After pouring, PDMS was degassed to eliminate bubbles introduced during mixing, then cured at 80°C for 60 min. After careful demolding and cropping of the chips, we created inlet and outlet ports at the ends of the flow channels using a 0.5 mm biopsy puncher (Shoney Scientific). Subsequently, chips and ports were washed with ethanol and rinsed with distilled water prior to bonding to 24 × 40 mm coverslips using a plasma cleaner. Bonded chips were immediately placed on a heating plate at 80°C for 2 min and incubated at 60°C overnight. One hour before cell loading, chips were plasma-activated and flushed with 20% polyethylene glycol to aid media flow and prevent cell attachment.

### Cell loading and experimental conditions

Exponential bacterial cultures were centrifuged at 4,000 rpm for 10 min, and the resulting pellet was resuspended in 200 µL of M9 medium. The suspension was injected through the outlet port of each flow channel, followed by the centrifugation of the chip at 1,500 rpm for 8 min to push bacteria into the growth wells. After inspecting the chip under the microscope to ensure that loading was successful, it was connected to inlet tubing running through a peristaltic pump (Ole Dich, Denmark) to generate a constant flow of 200 µL/h. At the outlet ports of the loaded chip, another set of tubing was connected to a waste container to collect the outflow. The chip was then placed in the microscope’s temperature-controlled incubation chamber (Pecon TempController 2000-1, set to 37°C) for the duration of each experiment.

### Microscopy and image acquisition

Time-lapse images were captured with a DS-Qi2 camera connected to an inverted microscope (Nikon Eclipse Ti2) equipped with a motorized stage and Perfect Focus System. Using a 100× oil objective and the NIS Elements software, once a chip was mounted in the microscope, we selected 12 fields for automated image capture in 2 min intervals. GFP and RFP fluorescence imaging (400 ms exposure and 8% intensity) was set to 10 min intervals to reduce phototoxic stress while providing sufficient data. Two separate replicates were performed for each strain.

### Image processing

After the experiments, images were first adjusted on ImageJ for chromatic shift correction and background subtraction of fluorescence images (rolling ball radius = 12 px). The tool Deep Learning for Time-lapse Analysis (DeLTA) (46) was used for automated image processing, which was previously trained on data from our experimental setup. This procedure was performed on the High-Performance Computing center at FU Berlin (47). Phase contrast images were used to perform cell segmentation and tracking. The DeLTA output was then converted into R data serialization files and visually inspected for quality control. The data for each bacterial lineage was run through a custom R script (version 2023.12.1+402) to correct obvious tracking errors, remove empty or only partially captured wells, and crop wells where severe tracking and segmentation errors occurred. Where necessary, further manual corrections were performed with the same custom R script.

### Quantification of bacterial growth

Elongation rates were used as a measure of cellular growth and fitness, because the rate at which the cell increases in length is crucial for its ability to double its length and divide. To measure elongation rates, each cell was followed from its “birth” until the point where it divided again, and the division interval was determined. Cell length was measured along the cell cycle. Based on these measurements and considering that single *E. coli* cells elongate exponentially (48), elongation rates were calculated as the exponential fit of the length increase of each cell over time. As mother and daughter cells originating from the same division were paired in the data, we were also able to determine morphological asymmetry by using length measurements after division to calculate the ratio of mother cell birth length to daughter cell birth length.

### Quantification of dispersed fluorescence and protein aggregates

To determine intracellular damage and protein aggregates, the first fluorescence image of each cell cycle was measured in terms of the brightness of each pixel within the area of the cell. GFP signal intensity was measured in arbitrary units, as fluorescence measurements depend on the microscope and its settings. The same settings were used in all experimental runs, prioritizing resolution at the bright end of the spectrum as we were interested in the areas of the cells with the most fluorescence. To determine the mean intensity of GFP fluorescence and thereby assess the relative amount of DnaK-indicated damage load per cell, an average value over all pixels in the cell was calculated.

From the DeLTA output, we obtained a pixel-by-pixel fluorescence profile along a transect running through the center of each cell, from the old pole to the new pole. In a cell with diffused fluorescence, transect measurements were relatively uniform. However, many cells displayed extreme spikes in fluorescence near the old poles, which could be detected as outliers in the transect data. By locating these outliers using the function findpeaks() in R, areas with a very high density of DnaK-msfGFP, representing aggregates, could be detected, and their absolute length, distance to the old pole, intensity peak, and mean fluorescence were determined with a custom R script. The ratio of aggregate length to cell length was calculated to gain information about the proportion of the cell that was taken by the aggregate. For visualization of the mean transect measurements over generations, generations with fewer than 10 cells were removed.

### Statistical analysis

All statistical analyses were performed in R (version 2024 4.3.3). Included in the analysis were mother cells, as well as their immediate new pole offspring (daughters). These pairs of old and new pole cells born in the same division formed a set of paired data points, each of them observed from its birth until its next division. Any cells that did not complete their division cycle and whose next division was therefore not captured were excluded from the analysis. In the same vein, cells that started their cell cycle before image capture began were also excluded. Mother cell age was determined both in the sense of replicative age (counted in generations, Fig. 1E and 2B, Fig. 4A and B) and chronological age (Fig. 1B through D and 2C through E, Fig. 3B and C), the latter with a resolution of 2 min, as this was the imaging interval. Generation and time count started with the start of imaging.

We used model selection based on AIC (Akaike’s Information Criterion) to differentiate the support of competing models (49). We interpret an ∆AIC >2 as substantially better support. Non-linear patterns were tested using GAM (R package mgcv with maximum likelihood estimation). Within each set of models compared, the best-supported model was evaluated for fitting and assumptions using diagnostic plots with the same statistical package. For analyzing mean fluorescence patterns (Fig. 1C and D), we used a negative binomially distributed error structure with an identity link function. For testing interactions between two continuous smoothing parameters (Fig. 1E), we used “ti” (tensor product interaction) rather than the otherwise implicit “s” smoother matrix. For comparison among density distributions in Fig. S1, we used two-sided two-sample Kolmogorov-Smirnov tests. When evaluating aggregate diameters (Fig. 2A), we were interested in estimating the linear growth and therefore used a linear model. Model assumptions were again evaluated using diagnostic plots. Detailed model estimates are provided in the Supplementary Materials. Data were standardized or normalized as required for visualization, but not for statistical testing. Where plots show binned data, bins represent mean ± standard deviation (SD), excluding bins that contained fewer than 10 data points.

## Data Availability

All data and supporting code are available from a public repository (Zenodo DOI: https://doi.org/10.5281/zenodo.15088910).

## SUPPLEMENTAL MATERIAL

Supplemental Material mbio.02050-25-s0001.docx Supplemental text, tables, and figures.

ASM does not own the copyrights to Supplemental Material that may be linked to, or accessed through, an article. The authors have granted ASM a non-exclusive, world-wide license to publish the Supplemental Material files. Please contact the corresponding author directly for reuse.

## References

[1] López-Otín C, Blasco MA, Partridge L, Serrano M, Kroemer G. 2013. The hallmarks of aging. Cell 153:1194–1217. doi:10.1016/j.cell.2013.05.039 23746838 PMC3836174

[2] Rose MR. 1991. Evolutionary biology of aging. Oxford University Press, New York.

[3] Kirkwood TBL. 1977. Evolution of ageing. Nature 270:301–304. doi:10.1038/270301a0 593350

[4] Coelho M, Lade SJ, Alberti S, Gross T, Tolić IM. 2014. Fusion of protein aggregates facilitates asymmetric damage segregation. PLoS Biol 12:e1001886. doi:10.1371/journal.pbio.1001886 24936793 PMC4061010

[5] Laney SR, Olson RJ, Sosik HM. 2012. Diatoms favor their younger daughters. Limnol Oceanogr 57:1572–1578. doi:10.4319/lo.2012.57.5.1572

[6] Ackermann M, Stearns SC, Jenal U. 2003. Senescence in a bacterium with asymmetric division. Science 300:1920. doi:10.1126/science.1083532 12817142

[7] Stewart EJ, Madden R, Paul G, Taddei F. 2005. Aging and death in an organism that reproduces by morphologically symmetric division. PLoS Biol 3:e45. doi:10.1371/journal.pbio.0030045 15685293 PMC546039

[8] Lindner AB, Madden R, Demarez A, Stewart EJ, Taddei F. 2008. Asymmetric segregation of protein aggregates is associated with cellular aging and rejuvenation. Proc Natl Acad Sci USA 105:3076–3081. doi:10.1073/pnas.0708931105 18287048 PMC2268587

[9] Proenca AM, Rang CU, Buetz C, Shi C, Chao L. 2018. Age structure landscapes emerge from the equilibrium between aging and rejuvenation in bacterial populations. Nat Commun 9:3722. doi:10.1038/s41467-018-06154-9 30213942 PMC6137065

[10] Steiner UK, Lenart A, Ni M, Chen P, Song X, Taddei F, Vaupel JW, Lindner AB. 2019. Two stochastic processes shape diverse senescence patterns in a single-cell organism. Evolution 73:847–857. doi:10.1111/evo.13708 30816556

[11] Winkler J, Seybert A, König L, Pruggnaller S, Haselmann U, Sourjik V, Weiss M, Frangakis AS, Mogk A, Bukau B. 2010. Quantitative and spatio-temporal features of protein aggregation in Escherichia coli and consequences on protein quality control and cellular ageing. EMBO J 29:910–923. doi:10.1038/emboj.2009.412 20094032 PMC2837176

[12] Coquel AS, Jacob JP, Primet M, Demarez A, Dimiccoli M, Julou T, Moisan L, Lindner AB, Berry H. 2013. Localization of protein aggregation in Escherichia coli is governed by diffusion and nucleoid macromolecular crowding effect. PLoS Comput Biol 9:e1003038. doi:10.1371/journal.pcbi.1003038 23633942 PMC3636022

[13] Partridge L, Barton NH. 1993. Optimality, mutation and the evolution of ageing. Nature 362:305–311. doi:10.1038/362305a0 8455716

[14] Ackermann M, Chao L, Bergstrom CT, Doebeli M. 2007. On the evolutionary origin of aging. Aging Cell 6:235–244. doi:10.1111/j.1474-9726.2007.00281.x 17376147 PMC2049046

[15] Chao L. 2010. A model for damage load and its implications for the evolution of bacterial aging. PLoS Genet 6:e1001076. doi:10.1371/journal.pgen.1001076 20865171 PMC2928801

[16] Evans SN, Steinsaltz D. 2007. Damage segregation at fissioning may increase growth rates: a superprocess model. Theor Popul Biol 71:473–490. doi:10.1016/j.tpb.2007.02.004 17442356 PMC2430589

[17] Schramm FD, Schroeder K, Jonas K. 2020. Protein aggregation in bacteria. FEMS Microbiol Rev 44:54–72. doi:10.1093/femsre/fuz026 31633151 PMC7053576

[18] Hipp MS, Kasturi P, Hartl FU. 2019. The proteostasis network and its decline in ageing. Nat Rev Mol Cell Biol 20:421–435. doi:10.1038/s41580-019-0101-y 30733602

[19] Linnik D, Maslov I, Punter CM, Poolman B. 2024. Dynamic structure of E. coli cytoplasm: supramolecular complexes and cell aging impact spatial distribution and mobility of proteins. Commun Biol 7:508. doi:10.1038/s42003-024-06216-3 38678067 PMC11055878

[20] Xiang Y, Surovtsev IV, Chang Y, Govers SK, Parry BR, Liu J, Jacobs-Wagner C. 2021. Interconnecting solvent quality, transcription, and chromosome folding in Escherichia coli. Cell 184:3626–3642. doi:10.1016/j.cell.2021.05.037 34186018

[21] Chao L, Chan CK, Shi C, Rang CU. 2024. Spatial and temporal distribution of ribosomes in single cells reveals aging differences between old and new daughters of Escherichia coli. eLife 12:RP89543. doi:10.7554/eLife.89543 39565213 PMC11578582

[22] Shi C, Chao L, Proenca AM, Qiu A, Chao J, Rang CU. 2020. Allocation of gene products to daughter cells is determined by the age of the mother in single Escherichia coli cells. Proc Biol Sci 287:20200569. doi:10.1098/rspb.2020.0569 32370668 PMC7282926

[23] Proenca AM, Tuğrul M, Nath A, Steiner UK. 2024. Progressive decline in old pole gene expression signal enhances phenotypic heterogeneity in bacteria. Sci Adv 10:eadp8784. doi:10.1126/sciadv.adp8784 39514668 PMC11546803

[24] Tyedmers J, Mogk A, Bukau B. 2010. Cellular strategies for controlling protein aggregation. Nat Rev Mol Cell Biol 11:777–788. doi:10.1038/nrm2993 20944667

[25] Chao L, Rang CU, Proenca AM, Chao JU. 2016. Asymmetrical damage partitioning in bacteria: a model for the evolution of stochasticity, determinism, and genetic assimilation. PLoS Comput Biol 12:e1004700. doi:10.1371/journal.pcbi.1004700 26761487 PMC4711911

[26] Proenca AM, Rang CU, Qiu A, Shi C, Chao L. 2019. Cell aging preserves cellular immortality in the presence of lethal levels of damage. PLoS Biol 17:e3000266. doi:10.1371/journal.pbio.3000266 31120870 PMC6532838

[27] Govers SK, Mortier J, Adam A, Aertsen A. 2018. Protein aggregates encode epigenetic memory of stressful encounters in individual Escherichia coli cells. PLoS Biol 16:e2003853. doi:10.1371/journal.pbio.2003853 30153247 PMC6112618

[28] Łapińska U, Glover G, Capilla-Lasheras P, Young AJ, Pagliara S. 2019. Bacterial ageing in the absence of external stressors. Phil Trans R Soc B 374:20180442. doi:10.1098/rstb.2018.0442 31587633 PMC6792439

[29] Dewachter L, Bollen C, Wilmaerts D, Louwagie E, Herpels P, Matthay P, Khodaparast L, Khodaparast L, Rousseau F, Schymkowitz J, Michiels J. 2021. The dynamic transition of persistence toward the Viable but Nonculturable state during stationary phase is driven by protein aggregation. mBio 12:e0070321. doi:10.1128/mBio.00703-21 34340538 PMC8406143

[30] Lemoine M. 2021. The evolution of the hallmarks of aging. Front Genet 12:693071. doi:10.3389/fgene.2021.693071 34512720 PMC8427668

[31] Hu C, Yang J, Qi Z, Wu H, Wang B, Zou F, Mei H, Liu J, Wang W, Liu Q. 2022. Heat shock proteins: biological functions, pathological roles, and therapeutic opportunities. MedComm (2020) 3:e161. doi:10.1002/mco2.161 35928554 PMC9345296

[32] Mayer MP. 2021. The Hsp70-chaperone machines in bacteria. Front Mol Biosci 8:694012. doi:10.3389/fmolb.2021.694012 34164436 PMC8215388

[33] Wang P, Robert L, Pelletier J, Dang WL, Taddei F, Wright A, Jun S. 2010. Robust growth of Escherichia coli. Curr Biol 20:1099–1103. doi:10.1016/j.cub.2010.04.045 20537537 PMC2902570

[34] Mortier J, Govers SK, Cambré A, Van Eyken R, Verheul J, den Blaauwen T, Aertsen A. 2023. Protein aggregates act as a deterministic disruptor during bacterial cell size homeostasis. Cell Mol Life Sci 80:360. doi:10.1007/s00018-023-05002-4 37971522 PMC11072981

[35] Baig UI, Bhadbhade BJ, Mariyam D, Watve MG. 2014. Protein aggregation in E. coli : short term and long term effects of nutrient density. PLoS One 9:e107445. doi:10.1371/journal.pone.0107445 25210787 PMC4161400

[36] Steiner UK. 2021. Senescence in bacteria and its underlying mechanisms. Front Cell Dev Biol 9:668915. doi:10.3389/fcell.2021.668915 34222238 PMC8249858

[37] Chai Q, Singh B, Peisker K, Metzendorf N, Ge X, Dasgupta S, Sanyal S. 2014. Organization of ribosomes and nucleoids in Escherichia coli cells during growth and in quiescence. J Biol Chem 289:11342–11352. doi:10.1074/jbc.M114.557348 24599955 PMC4036271

[38] Neurohr GE, Terry RL, Lengefeld J, Bonney M, Brittingham GP, Moretto F, Miettinen TP, Vaites LP, Soares LM, Paulo JA, Harper JW, Buratowski S, Manalis S, van Werven FJ, Holt LJ, Amon A. 2019. Excessive cell growth causes cytoplasm dilution and contributes to senescence. Cell 176:1083–1097. doi:10.1016/j.cell.2019.01.018 30739799 PMC6386581

[39] Mitsui Y, Schneider EL. 1976. Relationship between cell replication and volume in senescent human diploid fibroblasts. Mech Ageing Dev 5:45–56. doi:10.1016/0047-6374(76)90007-5 1263608

[40] Lengefeld J, Cheng C-W, Maretich P, Blair M, Hagen H, McReynolds MR, Sullivan E, Majors K, Roberts C, Kang JH, Steiner JD, Miettinen TP, Manalis SR, Antebi A, Morrison SJ, Lees JA, Boyer LA, Yilmaz ÖH, Amon A. 2021. Cell size is a determinant of stem cell potential during aging. Sci Adv 7:eabk0271. doi:10.1126/sciadv.abk0271 34767451 PMC8589318

[41] Demidenko ZN, Blagosklonny MV. 2008. Growth stimulation leads to cellular senescence when the cell cycle is blocked. Cell Cycle 7:3355–3361. doi:10.4161/cc.7.21.6919 18948731

[42] Taheri-Araghi S, Bradde S, Sauls JT, Hill NS, Levin PA, Paulsson J, Vergassola M, Jun S. 2015. Cell-size control and homeostasis in bacteria. Curr Biol 25:385–391. doi:10.1016/j.cub.2014.12.009 25544609 PMC4323405

[43] Tuğrul M, Steiner UK. 2025. Demographic consequences of damage dynamics in single-cell aging. Phys Rev Research 7. doi:10.1103/PhysRevResearch.7.013327

[44] Schramm FD, Schroeder K, Alvelid J, Testa I, Jonas K. 2019. Growth-driven displacement of protein aggregates along the cell length ensures partitioning to both daughter cells in Caulobacter crescentus. Mol Microbiol 111:1430–1448. doi:10.1111/mmi.14228 30779464 PMC6850343

[45] Yang D, Jennings AD, Borrego E, Retterer ST, Männik J. 2018. Analysis of factors limiting bacterial growth in PDMS mother machine devices. Front Microbiol 9:871. doi:10.3389/fmicb.2018.00871 29765371 PMC5938360

[46] Lugagne JB, Lin H, Dunlop MJ. 2020. DeLTA: automated cell segmentation, tracking, and lineage reconstruction using deep learning. PLoS Comput Biol 16:e1007673. doi:10.1371/journal.pcbi.1007673 32282792 PMC7153852

[47] Bennett L, Melchers B, Proppe B. 2020. CURTA a general-purpose high-performance computer at ZEDAT. Berlin, Germany Freie Universität Berlin.

[48] Robert L, Hoffmann M, Krell N, Aymerich S, Robert J, Doumic M. 2014. Division in Escherichia coli is triggered by a size-sensing rather than a timing mechanism. BMC Biol 12:17. doi:10.1186/1741-7007-12-17 24580833 PMC4016582

[49] Burnham KP, Anderson DR. 2004. Multimodel inference: understanding AIC and BIC in model selection. Sociol Methods Res 33:261–304. doi:10.1177/0049124104268644

